# Integrating gene regulatory pathways into differential network analysis of gene expression data

**DOI:** 10.1038/s41598-019-41918-3

**Published:** 2019-04-02

**Authors:** Tyler Grimes, S. Steven Potter, Somnath Datta

**Affiliations:** 1 University of Florida, Department of Biostatistics, Gainesville, 32611 USA; 2 University of Cincinnati, Department of Pediatrics, Cincinnati, 45229 USA

## Abstract

The advent of next-generation sequencing has introduced new opportunities in analyzing gene expression data. Research in systems biology has taken advantage of these opportunities by gleaning insights into gene regulatory networks through the analysis of gene association networks. Contrasting networks from different populations can reveal the many different roles genes fill, which can lead to new discoveries in gene function. Pathologies can also arise from aberrations in these gene-gene interactions. Exposing these network irregularities provides a new avenue for understanding and treating diseases. A general framework for integrating known gene regulatory pathways into a differential network analysis between two populations is proposed. The framework importantly allows for any gene-gene association measure to be used, and inference is carried out through permutation testing. A simulation study investigates the performance in identifying differentially connected genes when incorporating known pathways, even if the pathway knowledge is partially inaccurate. Another simulation study compares the general framework with four state-of-the-art methods. Two RNA-seq datasets are analyzed to illustrate the use of this framework in practice. In both examples, the analysis reveals genes and pathways that are known to be biologically significant along with potentially novel findings that may be used to motivate future research.

## Introduction

Next-generation sequencing (NGS) provides more information about the genomic involvement in cellular activity than past technologies. With microarrays, a set of genes must be specified in advance of the experiment. RNA-sequencing (RNA-seq) is open to the whole genome without any prior specification required. The technology uses short reads obtained from strands of RNA extracted from a sample of tissue cells. These reads can then be mapped to a reference genome, producing expression counts for each gene.

One application of RNA-sequencing is to investigate gene-gene associations. Genes do not work alone; they interact with each other in complex ways. Genes that are involved in the same biological pathway, controlled by the same transcription factor, or otherwise functionally related tend to have similar expression levels^1,2^. This is often referred to as the guilt-by-association principle, and it provides support for the use of gene expression data in reconstructing the underlying gene-gene association network^3,4^. Networks inferred from gene expression data are called gene co-expression networks (GCN); these are undirected graphs with nodes representing genes and edges representing gene-gene associations. The topology of the inferred network is used to make predictions about the genes^5–7^. For example, a hub gene in the network may be a transcription factor that regulates its connected genes^8^. Or, a connected component in the network might be a set of genes involved in a particular pathway or protein complex^9^.

A differential network analysis compares individual networks from different populations, or groups, to identify group-specific connections^10–12^. Differences in the topology of two networks may indicate differences in the underlying cellular activity. For example, the existence of differentially connected (DC) gene modules might indicate that various pathways have been rewired^13^.

Before conducting a differential network analysis, the researcher must decide on the measure of association to use: What do we mean by gene-gene association? In many modern methods, four of which are reviewed later, the definition of association is made for us. However, it’s important to make this choice based on the context of the biological question at hand. Different measures can lead to vastly different results, and this choice should be out in front of the analysis, not hidden away in a black box.

Probably the most common association measure is the well-known Pearson correlation coefficient^14,15^. This measure provides marginal, linear associations between genes. That is, each pair of genes is considered alone, ignoring the presence of all remaining genes. The resulting networks will often be very dense, and the natural interpretation of the edges - that of a direct connection - is not valid; the edges represent marginal, not direct or causal connections.

Conversely, the partial correlation does reveal direct connections. It is a measure of conditional, linear associations. A thorough review of partial correlations is available^16^. If the gene expression profiles follow a multivariate Gaussian distribution, then two genes have non-zero partial correlation if and only if they are conditionally dependent given the other genes^16^. The resulting network of conditionally dependent nodes is often called a Gaussian graphical model (GGM). This model is particularly appealing because it can be used to reframe the problem of estimating partial correlations in terms of maximizing a penalized log-likelihood function. In the latter optimization setting, desired properties of the networks can be enforced by restricting the solution space or by constructing an appropriate penalty. Researchers have exploited this flexibility, resulting in a diverse literature of analyzing gene expression using GGMs^17–22^.

Other measures may attempt to capture nonlinear associations between genes. This can be done using association measures based on information theory or non-parametric models^23–27^. More recently, a measure that directly models the count data generated from RNA-seq experiments was proposed^28^.

The incorporation of pathway information has been shown to improve performance in differential expression (DE) analyses. Researchers have used KEGG pathways^29^ to construct a Markov random field (MRF) that improves performance in finding DE genes^30^. Others used KEGG pathways to inform a spatially correlated mixture model for DE analysis^31^. Reactome pathways^32^ have also been utilized in DE analysis; in one example altered pathways in lung adenocarcinoma and colon cancer were identified^33^. The integration of pathway information into DE analysis is an on-going area of research^34–36^.

In DC analyses, pathway information is often used but in an ad hoc way. New methods of differential network analysis are often demonstrated in an application to a particular pathway^27^, a set of pathways^15,20^, or after filtering genes through univariate analyses^19^. A gene expression profile can contain thousands of genes, so it is usually necessary to focus on a smaller subset of genes to comply with computational limitations.

This report proposes a framework for integrating known genetic pathways into a differential network analysis of two populations. The framework allows any association measure to be used, and a general measure for differential connectivity is considered. Statistical significance is evaluated through a permutation testing procedure. The methodology is implemented in R and is available on GitHub at https://github.com/tgrimes/dnapath.

A simulation study assesses the gain in performance when incorporating pathway information. We also consider the problem that in reality our knowledge of pathways is incomplete; any given pathway may have missing genes, genes wrongly included, or both. The simulation study considers the performance under this type of pathway misspecification. We also compare this approach to four modern methods: DGCA^15^, DINGO^20^, INDEED^19^, and JDINAC^27^. The application of this framework for exploratory analysis is illustrated on two RNA-seq datasets.

## Methods

### Problem formulation

A differential network analysis is considered when gene expression samples are collected from two distinct populations or “groups”. The analysis compares the set of inter-connections of genes between the groups. Additionally, the existence of gene pathways is assumed. For our purpose, a pathway is considered to be a subset of genes that act together to carry out a specific molecular function.

Let ${X}^{k}\in {{\mathbb{R}}}^{{n}_{k}\times m}$ denote an observed gene expression profile containing *m* genes for *n*_*k*_ samples in group $k\in \{1,2\}$. The rows and columns of *X*^*k*^ are indexed by ${X}_{i\cdot }^{k}\in {{\mathbb{R}}}^{m}$ and ${X}_{\cdot j}^{k}\in {{\mathbb{R}}}^{{n}_{k}}$, respectively. The samples ${X}_{i\cdot }^{k}$ are assumed independent and identically distributed, conditioned on the group *k*. Let ${\mathscr{G}}\subset {\mathscr{P}}(\{1,\ldots ,m\})$ denote a collection of pathways of interest, where ${\mathscr{P}}$ is the power set. $G\in {\mathscr{G}}$ contains the indices for the genes in pathway *G*, and ${X}^{k}(G)\in {{\mathbb{R}}}^{{n}_{k}\times |G|}$ is the observed expression profile with columns subset on those genes.

For a given pathway $G\in {\mathscr{G}}$, our interest is in estimating the gene-gene association networks for both groups $k=1,2$, which is represented by the real symmetric matrix ${S}^{k}(G)\in {{\mathbb{R}}}^{|G|\times |G|}$, where |*G*| denotes the cardinality of the set *G*. To compare the network between the groups, we propose a nonnegative measure, ${\delta }_{E}({S}^{1}(G),{S}^{2}(G))$. This measure determines the differential connectivity of a set $E\subset  {\mathcal E} (G)$ of connections, where ${\mathcal E} (G)=\{(i,j)|i,j\in G,i < j\}$ is the set of all possible connections in the network. A value of zero indicates no change in the connections contained in *E*. Statistical significance is assessed through a permutation testing procedure.

To simplify notation, *X*^*k*^(*G*), ${\mathcal E} (G)$, *S*^*k*^(*G*) may be written as *X*^*k*^, ${\mathcal E}$, and *S*^*k*^ with the dependence on *G* inferred from the context. Bold font **X** will denote a random variable and regular font *X* will denote an observation. Without loss of generality, the columns of *X*^*k*^ are assumed to be centered, such that ${\sum }_{i}\,{X}_{ij}^{k}=0$ for $k=1,2$ and $j=1,\ldots ,m$.

### Differential network analysis

The proposed framework for conducting a differential network analysis is summarized in the following steps, with details given in the proceeding sections.

1. Given a pathway *G*, compute the estimated association networks, ${\hat{S}}^{1}$ and ${\hat{S}}^{2}$, for the two groups.
2. Evaluate the differential connectivity scores, *δ*_*E*_, for each $E\in  {\mathcal E} (G)$ of interest.
3. Assess the statistical significance of these scores using a permutation testing procedure.
4. Repeat steps 1–3 for each pathway $G\in {\mathscr{G}}$.

#### Estimation of association networks

The first step is to estimate the gene-gene association network within each group. There is no restriction on the meaning of “association” in the problem formulation other than symmetry. This choice of which association measure to use is left to the practitioner and will likely depend on the biological problem being studied, the amount of data available, and other factors. In this section, we review a variety of approaches that can be considered.

The most common approach is to use simple pair-wise correlations. Let ${\rm{\Sigma }}=E({{\bf{X}}}_{1\cdot }{({{\bf{X}}}_{1\cdot })}^{T})$ denote the covariance matrix between genes in a fixed group, where **X**_1·_ is a (*m*-dimensional) random variable. Here, the first row of *X* can be taken without loss of generality, since we assume that observations are i.i.d. within the group. The marginal correlation between genes *i* and *j* is,

1 $${r}_{ij}={{\rm{\Sigma }}}_{ij}/{[{{\rm{\Sigma }}}_{ii}{{\rm{\Sigma }}}_{jj}]}^{1/2}.$$

These correlations can be estimated using the sample covariance matrix $\hat{{\rm{\Sigma }}}={(n-1)}^{-1}{(X)}^{T}X$, where *n* is the number of observations in the group. Alternatively, a robust estimator can be used, such as Spearman’s rank-based correlation, Kendall’s correlation, and Gaussian rank correlation^37^.

Marginal correlation is used in weighted correlation network analysis (WGCNA)^38^, which is motivated from the scale-free topology observed in biological networks. In WGCNA, the estimates ${\hat{r}}_{ij}$ are computed for each pair of genes and soft thresholding is used to send smaller values towards zero.

2 $${\hat{S}}_{ij}=|{\hat{r}}_{ij}{|}^{\beta },$$

where $\beta \ge 1$ is the soft-thresholding parameter. This is in contrast to hard thresholding,

3 $${\hat{S}}_{ij}=\{\begin{array}{cc}{\hat{r}}_{ij} & {\rm{if}}\,|{\hat{r}}_{ij}| > \gamma \\ 0 & {\rm{otherwise}}\end{array},$$

in which correlations below a hard threshold parameter, $\gamma  > 0$, are set to zero.

An alternative measure is partial correlation, which gives the conditional correlation rather than marginal. That is, the linear association between two genes is considered after accounting for the presence of the remaining genes in the pathway. Let ${\rm{\Omega }}={{\rm{\Sigma }}}^{-1}$ denote the inverse of the covariance matrix, also called the precision matrix. Partial correlations between genes can be computed from the precision matrix by

4 $${\rho }_{ij}=-\,{{\rm{\Omega }}}_{ij}/{[{{\rm{\Omega }}}_{ii}{{\rm{\Omega }}}_{ii}]}^{1/2}.$$

If a gene expression profile follows a multivariate normal distribution, then zero partial correlation implies conditional independence of genes; this is called the Gaussian graphical model (GGM). In this model, the precision matrix can be estimated from the log-likelihood function by

5 $$\hat{{\rm{\Omega }}}={{\rm{argmax}}}_{{\rm{\Omega }}\succ 0}\{\,\mathrm{log}\,{\rm{\det }}({\rm{\Omega }})-{\rm{tr}}({\rm{\Omega }}\hat{{\rm{\Sigma }}})\},$$

where ${\rm{\Omega }}\in {{\mathbb{R}}}^{m\times m}$ and ${\rm{\Omega }}\,\succ \,0$ denotes the set of positive definite matrices. Alternatively, a penalized log-likelihood function can be used to enforce desirable properties of the network. For example, the graphical lasso enforces sparsity in the network by shrinking some off-diagonal elements to zero through an *L*_1_ penalty^39^.

The problem of estimating large precision matrices has been studied extensively; a review of estimation procedures implemented in R is available^40^, as well as an overview with a concentration on rank based and factor model based methods^41^.

We estimate partial correlations using a shrinkage approach^42^. This estimator was chosen because of its favorable computational properties^42^. The method uses a mixture of a low-dimensional estimate, $\hat{T}$, and unconstrained estimate, $\hat{{\rm{\Sigma }}}$. In this case, $\hat{{\rm{\Sigma }}}$ is the sample correlation matrix for group *k*, and $\hat{T}$ is the diagonal matrix with ${\hat{T}}_{ii}={\hat{{\rm{\Sigma }}}}_{ii}$, for $i=1,\ldots ,m$, and zeros on the off-diagonal. The shrinkage estimator, ${\hat{{\rm{\Sigma }}}}^{\ast }$, is defined as the linear combination

$${\hat{{\rm{\Sigma }}}}^{\ast }=\lambda \hat{T}+(1-\lambda )\hat{{\rm{\Sigma }}},$$

where *λ* is a shrinkage parameter. It has been shown^43^ that there exists an analytical solution for the optimal *λ*, denoted by λ*, which minimizes $\parallel {\rm{\Sigma }}-\hat{{\rm{\Sigma }}}{\parallel }_{F}^{2}$, where *F* denotes the Frobenious norm. The solution λ* for various choices of low-dimensional spaces containing $\hat{T}$ have been derived^42^. In this study, the shrinkage target is the space containing uncorrelated genes allowing for unequal variances. The corresponding optimal shrinkage parameter is

$${\lambda }^{\ast }=\sum _{i\ne j}\widehat{Var}({\hat{{\rm{\Sigma }}}}_{ij})/\sum _{i\ne j}\,{\hat{{\rm{\Sigma }}}}_{ij}.$$

The estimate for the precision matrix is obtained from the inverse of the shrinkage covariance estimate, $\hat{{\rm{\Omega }}}={({\hat{{\rm{\Sigma }}}}^{\ast })}^{-1}$.

#### Differential connectivity score

The differential network analysis measures the change in a set of connections $E\subset  {\mathcal E} (G)=\{(i,j)|i,j\in G,i < j\}$ for a given pathway $G\in {\mathscr{G}}$. We generalize the differential connectivity score proposed in earlier work^44^ by the *p*-norm of the difference in connectivity scores in *E*:

6 $${\delta }_{E}({S}^{1},{S}^{2})={(\frac{1}{|E|}\sum _{(i,j)\in E}|{S}_{ij}^{1}-{S}_{ij}^{2}{|}^{p})}^{1/p},$$

where $p\ge 1$ is fixed and $| {\mathcal E} |=|G|(|G|-1)/2$ denotes the number of possible connections in the pathway *G*; the weight 1/|*E*| accounts for the varying sizes of pathways. For $0 < p < 1$, the same expression is used to define *δ*_*E*_ but with the 1/*p* exponent removed.

The elements in *E* can be chosen to test different components of the network: for differential connectivity of the whole pathway, $E= {\mathcal E} (G)$; for differential connectivity of gene $i\in G$, $E=\{e\in  {\mathcal E} (G)|i\in e\}$; and for the differential connectivity of a single association between genes *i* and *j* in *G*, with $i < j$, *E* is the singleton $E=\{(i,j)\}$. Note, in this last case the choice of *p* is inconsequential since the sum in *δ*_*E*_ is over a single element.

#### Tests for significance

For a given set of connections *E*, we consider the hypothesis test,

$$\begin{array}{lll} & {H}_{0}:{S}^{1}{(G)}_{ij}={S}^{2}{(G)}_{ij}, & {\rm{for}}\,{\rm{every}}\,(i,j)\in E,\\ {\rm{vs}} & {H}_{1}:{S}^{1}{(G)}_{ij}\ne {S}^{2}{(G)}_{ij}, & {\rm{for}}\,{\rm{some}}\,(i,j)\in E,\end{array}$$

with test statistic $d={\delta }_{E}({\hat{S}}^{1}(G),{\hat{S}}^{2}(G))$. The null hypothesis says that the connections in *E* are consistent across both groups. Under this null, the group labels, $k\in \{1,2\}$, for each observation are immaterial when computing *S*^*k*^(*G*). This sets up a permutation testing procedure that can be used to estimate a p-value for *d* under the null, whereby permutations of the group labels are used, i.e. the observations are shuffled between groups. The total number of distinct permutations will often be quite large even for moderate sample sizes. In this case, the exact p-value is estimated from *B* randomly sampled permutations. This estimated p-value is adjusted to ensure it is positive^45^. The following algorithm outlines the permutation procedure.

1. Compute ${d}_{0}={\delta }_{E}({\hat{S}}^{1},{\hat{S}}^{2})$ on the original sample. Set $i=1$
2. Set $$X=[\begin{array}{c}{X}^{1}\\ {X}^{2}\end{array}]\in {{\mathbb{R}}}^{({n}_{1}+{n}_{2})\times p}$$.
3. Permute the rows of *X* to obtain a permuted matrix X*. Use the first *n*_1_ rows for ${X}^{1\ast }$ and the remaining *n*_2_ rows for ${X}^{2\ast }$.
4. Estimate the association networks ${\hat{S}}^{\mathrm{1\ast }}$ and ${\hat{S}}^{\mathrm{2\ast }}$ using the permuted samples ${X}^{\mathrm{1\ast }}$ and ${X}^{\mathrm{2\ast }}$.
5. Compute $${d}_{i}={\delta }_{E}({\hat{S}}^{1\ast },{\hat{S}}^{2\ast })$$.
6. Increment $$i=i+1$$.
7. Repeat steps 3–6 for a total of *B* times.
8. Return $(b+1)$/$(B+1)$ as the estimated p-value, where $b={\sum }_{i=1}^{B}\,I({d}_{0}\le {d}_{i})$.

In the case of multiple hypothesis testing, i.e. multiple sets *E* to be tested within a pathway, the Westfall-Young step-down p-values can be used to help control the false discovery rate^46,47^. This procedure monotonizes the p-values with respect to the original test statistics; larger differential connectivity scores will always correspond with lower p-values. The algorithm to compute these monotonized p-values is provided in the Supplementary Materials section S1.

### Simulation studies

Two simulation studies are performed. The first is used to assess the network-wide performance of the proposed framework in detecting DC pathways, DC genes, and DC edges when pathway information is used. The second study compares the performance within a single pathway to four alternative methods, including DGCA^15^, DINGO^20^, INDEED^19^, and JDINAC^27^.

DGCA is available the CRAN R package ‘DGCA’ (version 1.0.1); DINGO is implemented in the CRAN R package ‘iDINGO’ (version 1.0.2); an R package for INDEED is available on GitHub at https://github.com/ressomlab/INDEED (version 0.99.19); and there is no package for JDINAC, but the R source code is available on GitHub at https://github.com/jijiadong/JDINAC (accessed on November 27, 2018). The default settings for each of these methods are used. In DGCA, several options for multiple testing correction are available; we use the permutation option with 100 permutations. In DINGO, we lowered the suggested number of permutations from 100 to 20 due to computation constraints. For INDEED, the sparsity parameters are selected by cross validation using one standard error rule, and the number of permutations is set to 1000 as recommended. In JDINAC, the weight threshold is set to 4, with 10 splits and 5 folds used.

Both studies follow the same overall procedure for simulating data. Two GGMs are created to represent the underlying gene-gene network for two distinct groups. In the first study, the networks contain 500 genes, 9 of which are hub genes, and 20 pathways. In the second study, the networks contain 100 genes with one hub gene and a single pathway. A network is generated as follows:

1. For each pathway, generate a random pathway size from a negative binomial distribution with mean 20 and standard deviation 10.
2. Initialize each pathway by randomly selecting nodes from the network to populate the pathway, then generate a scale-free structure to connect these nodes using the Watts-Strogatz method^48^.
3. From the union of nodes selected for the pathways, randomly select nine to rewire as hub nodes. In each pathway containing one of these hub nodes, the hub node has a 50% chance of being connected to each node in that pathway.
4. At this point the creation of the first network is complete. The second network is initially identical to the first with the following modifications: one third of the hub nodes are turned off (all connections removed), another third are rewired, and the remaining third are left unchanged. In addition, 2.5% of the non-hub nodes are rewired.

These steps result in two distinct graphs (network structures) with several differentially connected genes. A graphical representation of the differential network (and the individual pathways) that was generated for the simulation is shown in Supplementary Figs S1 and S2.

The edges in these graphs correspond to nonzero partial correlations, i.e. nonzero values in the precision matrix, ${\rm{\Omega }}$. The next step is to generate values for these partial correlations.

1. For each network $k\in \{1,2\}$, the precision matrix, ${{\rm{\Omega }}}^{(k)}$, is initialized as an identity matrix.
2. The non-zero partial correlations in the lower triangle of ${{\rm{\Omega }}}^{(k)}$ are generated from a uniform distribution on $(\,-\,1,-\,0.5)\cup (0.5,1)$. Edges common to both networks are set to have the same partial correlation. The entries in the upper-triangle are set to ensure symmetry.
3. Positive definiteness is enforced by increasing the diagonal entries by $c=\,{\rm{\max }}({c}^{(1)},{c}^{(2)})$, where ${c}^{(k)}=({\lambda }_{(m)}^{(k)}{10}^{-1}-{\lambda }_{(1)}^{(k)})I({\lambda }_{(1)}^{(k)} < {\lambda }_{(m)}^{(k)}{10}^{-1})$, and ${\lambda }_{(1)}^{(k)}$ and ${\lambda }_{(m)}^{(k)}$ are the smallest and largest eigenvalues of ${{\rm{\Omega }}}^{(k)}$, respectively. The value *c*^(*k*)^ controls the condition number of ${{\rm{\Omega }}}^{(k)}$ and ensures numerical stability when computing its inverse^49^, and the maximum over both networks is used to ensure that common edges have the same partial correlations after the adjustment.

Performance is assessed through sensitivity, specificity, true discovery rate (TDR), true non-discovery rate (TNDR), F1 score, and Matthews correlation coefficient (MCC). Let TP, TN, FP, and FN denote the number of true positives, true negatives, false positives, and false negatives, respectively. Then, each performance measure is defined as follows:

$$\begin{array}{rcl}{\rm{Sensitivity}} & = & TP/(TP+FN),\\ {\rm{Specificity}} & = & TN/(TN+FP),\\ {\rm{TDR}} & = & TP/(TP+FP),\\ {\rm{TNDR}} & = & TN/(TN+FN),\\ {\rm{F}}1\,{\rm{score}} & = & 2\,{({({\rm{Sensitivity}})}^{-1}+{({\rm{TDR}})}^{-1})}^{-1},\\ {\rm{MCC}} & = & \frac{TP\times TN-FP\times FN}{\sqrt{(TP+FP)\,(TP+FN)\,(TN+FP)\,(TN+FN)}}.\end{array}$$

The F1 score is the harmonic mean of sensitivity and TDR. It conveys the balance between detecting many true differential connections while keeping the false discovery rate low. This measure completely ignores the number of true negatives, which can be quite large when dealing with sparse networks. MCC is a summary measure that reflects the overall performance; it includes the number of true negatives without being heavily influenced by the large imbalance of positives to negatives^50^.

The performance under misspecified pathways is also assessed. Pathway misspecification is simulated by introducing errors into our pathway knowledge; in particular, we mimic the scenario that pathways have both missing genes and genes wrongly included. At 100% knowledge, the pathway information used for analysis is identical to the true pathways used to generate the data. For misspecification, we introduce errors into the pathway information by replacing a portion of genes in each pathway. For example, with 90% pathway knowledge, 10% of the genes in each pathway are removed and replaced with genes outside of the pathway. This perturbed pathway information is then used in the differential network analysis as usual, and the effect on performance can be evaluated.

When no pathway information is being considered, the differential network is carried out using a single “pathway” containing all genes in the network.

### RNA-seq datasets

#### Craniofacial data of E14.5 mice

RNA-seq data from eight palatal regions of E14.5 (embryonic day 14.5) mice are analyzed^51^. E14.5 is a time during development when the palatal shelves are fused together. The eight regions are organized into four pairs that are analyzed in turn; these include the anterior and posterior domain of the Lateral, Medial, Nasal, and Oral compartments.

The differential network analysis attempts to identify changes in gene-gene interactions between the two domains; this type of behavior may, for example, be indicative of transcription factors involved in orchestrating gene expression during this phase of craniofacial development^52,53^. However, this investigation must be considered as exploratory, and the top DC genes may be considered for further validation.

The RNA-seq BAM files are available from the FaceBase Consortium^54,55^ repository using accession numbers FB00000753.01, FB00000754.01, FB00000757.01, FB00000758.01, FB00000761.01, FB00000762.01, FB00000765.01, and FB00000767.01. The reads are aligned and annotated using the mm9 reference genome from the UCSC Genome Browser^56^. There were 21585 genes mapped that had both an Entrez gene ID and MGI symbol. Genes on the Y chromosomes were removed, leaving 21094 genes. The read counts were normalized using transcripts per kilo-base million (TPM) normalization^57^ followed by a ${\mathrm{log}}_{2}(1+x)$ transformation. Genes with zeros in more than one third of the samples were filtered out; this threshold was set fairly low since only 3–4 samples are available per region.

#### Neuroblastoma tumor samples

Data for 498 neuroblastoma tumors are analyzed. These data contain heterogeneous gene expression profiles with diverse clinical outcomes. It is assumed that the differences in patient outcomes are largely a consequence of the differences in the somatic mutations present in their tumors^58,59^. Mutations in a gene may alter the function of the gene product, which may in turn alter the interaction of the gene with other genes^60^. The goal of a differential network analysis is to compare two clinically distinct subgroups of tumors and identify any differentially connected genes. This produces a set of postulated genes that may explain the disparity in prognosis. Further investigation into specific connections or altered pathways may contribute to improving risk stratification or motivate potential therapeutic targets for new treatments^61,62^.

The neurobalstoma data are obtained from the GEO database with accession number GSE49711^63,64^. Several clinical variables for each patient are available, including a high-risk (HR) indicator. We use this label to partition patients into two groups: HR versus non-HR. Normalization of the RNA-seq data was previously performed^63^, which is retained, unmodified in this analysis. There are 17115 genes mapped with both an Entrez gene ID and HGNC symbol. Incidentally, all genes had non-zero expression in at least 20% of the samples; no additional filters to remove lowly expressed genes were applied.

### Reactome pathway database

Pathway information is obtained for both mice (*mus musculus*) and humans (*homo sapiens*) from the Reactome database^32^. Only pathways containing between 10 and 100 genes were considered. Some of the pathways have significant overlap, sometimes differing by only one gene. This is due to the fact each biological process is broken down into separate events in Reactome, and at the lowest levels a series of events will often contain the same sets of genes. In our analysis, these specialized events containing a significant overlap of genes will be represented by a single pathway. This grouping can be implemented by hierarchical clustering using 1 minus the Jaccard Index as a distance measure, $d(A,B)=1-|A\cap B|/|A\cup B|$. By trimming the resulting dendrogram fairly low, for example at 0.1, the specialized events will be grouped together, and the higher-level pathways will remain separated. A concise review of hierarchical clustering is available^65^.

After clustering, we obtain 918 distinct pathways for *mus musculus* and 1160 distinct pathways for *homo sapiens*. In application, it is desirable to further remove any inactive pathways to help avoid spurious associations. This can be done in two ways: using domain knowledge to remove irrelevant pathways, or using the gene expression profiles by assuming that pathways containing many unexpressed genes are likely to be inactive. In this study, we take the latter approach - pathways containing over 20% unexpressed genes are considered inactive. Unexpressed genes include those that have zero counts in all samples, have been previously filtered out, or are otherwise not present in the gene expression profile. A threshold of 50% was also considered, but no substantial changes in the top results were found.

### Accession codes

The craniofacial data are available from the FaceBase Consortium using accession numbers FB00000753.01, FB00000754.01, FB00000757.01, FB00000758.01, FB00000761.01, FB00000762.01, FB00000765.01, and FB00000767.01; the neuroblastoma data are available on the GEO database with accession number GSE49711.

## Results

### Simulation study

**Figure 1:**
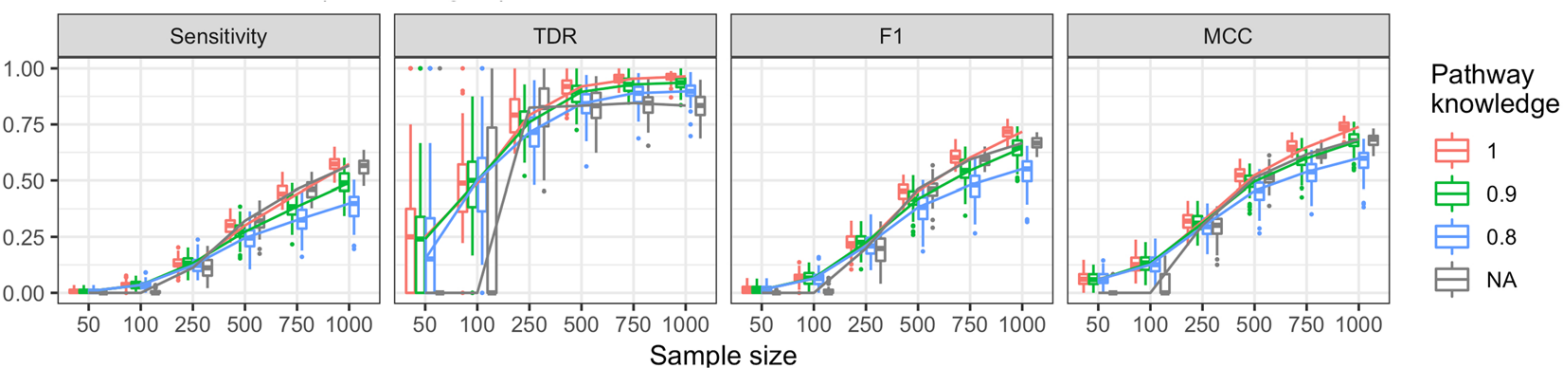
Simulation results from 100 generated datasets testing for DC edges based on partial correlation. Monotonized p-values from 100 permutations were compared to a 0.05 significance threshold. Results show the differential network analysis conducted without any pathway information (black), with complete pathway information (red), with 90% correct pathway information (green), and 80% correct pathway information (blue). The specificity and TNDR were approximately 1 for each setting and sample size, hence those graphs are not shown here.

The first simulation study is used to assess the performance in detecting DC edges, DC genes, and DC pathways. Figure 1 provides the results for DC edges using monotonized p-values with partial correlation as the association measure. Results for other settings can be found in the supplementary materials.

The incorporation of pathway information into the analysis provides a boost in sensitivity when dealing with smaller samples ($n < 250$). Even if the pathways are partially misspecified, the overall performance remains comparable. In Fig. 1, the gap in sensitivity between complete pathway knowledge versus partial knowledge begins to widen with large samples ($n > 500$); for other simulation settings, for example if Pearson correlations are used instead of partial correlations, this gap can occur earlier. However, in all settings considered, the lack of complete pathway knowledge maintained high specificity (i.e. low type-I error rate) and true discovery rate.

The proposed framework has a parameter *p* for the norm used in the DC score. A sensitivity analysis on the choice of *p* is carried out. The simulation results suggest that the performance increases with larger *p* but eventually reaches a plateau. For detecting DC genes with $n=250$ observations in both groups, the plateau is reached at $p=2$ (see Supplementary Fig. S5). With $n=1000$, this point is shifted to a point $p > 2$, but increasing *p* also expands the disparity between complete and partial pathway knowledge (see Supplementary Fig. S6). Based on these results, a robust choice appears to be $p=2$, which results in an *L*_2_ norm.

**Figure 2:**
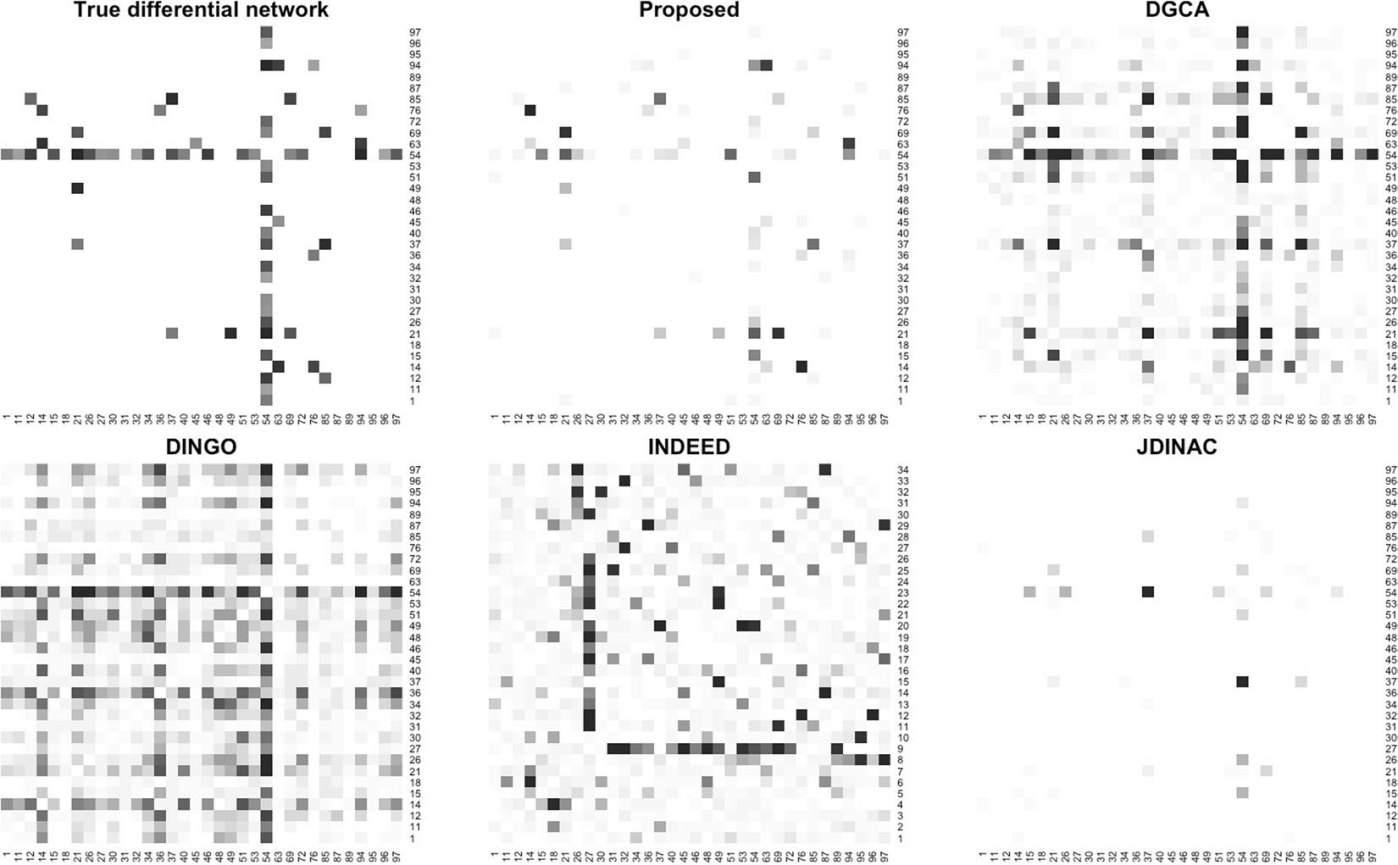
The average differential networks estimated by each method based on 20 generated samples of size $n=250$. The cells are shaded based on how frequently the edge was identified as differentially connected in the 20 samples; darker cells indicate higher frequency. The true differential network is based on the absolute difference in the precision matrices of the two populations.

In the second simulation study, the proposed method (using monotonized p-values) is compared to four modern approaches: DGCA^15^, DINGO^20^, INDEED^19^, and JDINAC^27^. Figure 2 shows the average estimated differential network by each method on 20 random samples generated from a single pathway.

It is clear from Fig. 2 that not all methods are estimating the same associations, so it is unreasonable to assume they are all directly comparable. The top-left matrix shows the true differential network, but this is with respect to partial correlations. If a different association measure was considered, then this “true” differential network would change. For partial correlations, we see that the proposed method is able to correctly identify DC edges without many false discoveries.

The DGCA method is based on pair-wise correlation. The estimated network from DGCA appears to be noisy, but that is only because it is being compared to a “true” network of partial correlations. If the true differential network is constructed using correlation instead, we find that many of the edges that currently appear to be false discoveries are actually true differential correlations (see Supplementary Fig. S7).

On the other hand, DINGO and INDEED are both based on partial correlation, so they should be relatively comparable to the proposed method. DINGO attempts to find group-specific conditional dependencies by decomposing the GGM into global and local (group-specific) components. In this simulation, it has a higher sensitivity but also a much higher false discovery rate. INDEED uses graphical lasso to estimate the precision matrix for each group. We would expect the performance to be comparable, but surprisingly most of the DC edges identified are false discoveries.

JDINAC is a non-parametric approach that is designed to detect differences in nonlinear associations. Since this method is not based on partial correlations, a direct comparison to the proposed method may not be reasonable. However, since this simulation is based on a GGM, the changes in conditional dependencies detected by JDINAC are comparable to the changes in partial correlations detected by the proposed method. In this setting, JDINAC has a very low sensitivity but high TDR. A more fair comparison could be made if a measure of nonlinear association was used in the proposed method, but this doesn’t escape the fact that the two methods will still be operating under different definitions of associations, and any comparison between them may inherently favor one over the other.

### Application to RNA-seq datasets

Two RNA-seq datasets are used to illustrate how an exploratory analysis can be carried out using the proposed framework. In both examples, a single pathway is analyzed in depth; a similar analysis can be conducted for any of the significantly DC pathways. Ideally, the practitioner will use domain knowledge when selecting from the list of DC pathways those that should be investigated further. To help guide the selection, other criteria can be applied, such as requiring the pathways to also be highly expressed (compared to the average pathway, for example) or differentially expressed between groups.

In both of the real datasets, the partial correlation is chosen for the association measure since we are interested in identifying changes in the direct connections between genes. Based on the simulation study results, we set $p=2$ for the DC scores. Monotonized p-values are used to identify DC pathways and DC genes.

#### Craniofacial data of E14.5 mice

Within each of the four palatal compartments (lateral, medial, nasal, and oral), we explore the differential connections in gene-gene associations between the anterior and posterior domains. The top DC pathways and DC genes for each compartment are shown in Supplementary Tables S1 and S2, respectively. For the permutation test, the smallest obtainable p-value with the given sample size ($n=3$ in each group) is 0.1, hence this is the threshold used for significance. These DC pathways and DC genes are also required to be highly expressed, i.e. among the top 50% in terms of expression level; this helps us identify the most active pathways. Furthermore, pathways or genes that are also significantly DE at the 0.1 significance level are moved to the top of the list. In the remainder of this section, results for the lateral compartment are discussed.

**Figure 3:**
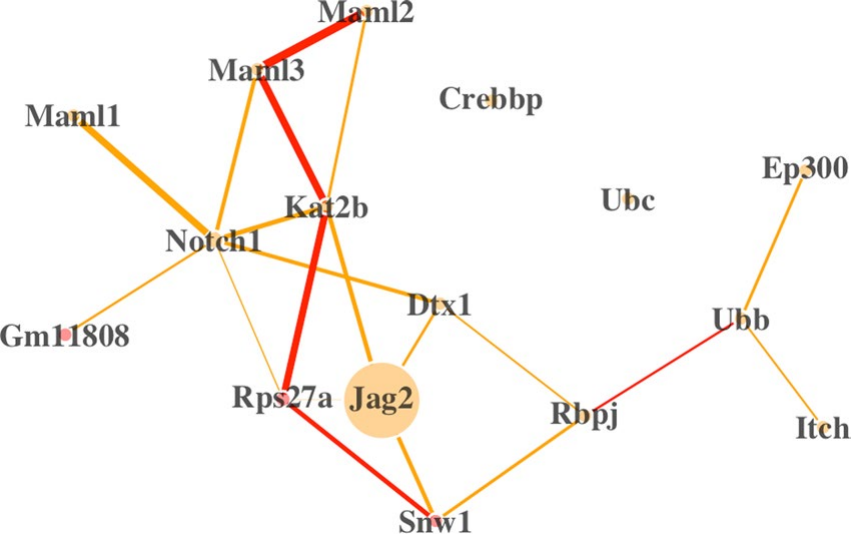
The differential network for the pathway “Signaling by NOTCH1” in the lateral compartment. Tan edges are associations that are stronger in the anterior domain, and red edges are stronger in the posterior domain. Edges are scaled proportional to the DC score, and nodes are scaled proportional to the fold change in expression between the two domains. Here, Jag2 is the only gene that is significantly DE. If the monotonized p-values for edges are preferred, then two edges remain significant: Maml2-Maml3 and Notch1-Maml1.

One of the top differentially connected Reactome pathways is Signaling by NOTCH1, which is a highly conserved pathway in developmental biology^66^. Within this pathway, the receptor gene Jag2 is differentially expressed - there is a 3.0 fold change in expression from the anterior domain to the posterior. Furthermore, we find supporting evidence that Jag2 may also be differentially connected; it appears to lose many of its connections to other genes in the posterior domain (see Fig. 3). On the other hand, the regulator gene Kat2b, though not differentially expressed, is differentially connected and is acting in consort with different sets of genes in the two domains. Jag2 is a gene that has been linked to the development of a cleft palate in mice^67^, and Jag2-Notch1 signaling has been shown to be a regulator in palate development^68^. Our findings suggest that Kat2b may also have an important, undiscovered role in the fusion of the palatal shelves. Indeed, in a very recent publication Kat2a and Kat2b are suggested to be epigenetic regulators required for craiofacial bone and cartilage growth and differentiation^69^.

Five of the top ten DC genes between the anterior and posterior domains of the lateral region have been shown to be involved in craniofacial development: Ep300 is linked to abnormal facial morphology^70^; Ngfr is associated with abnormal molar crown morphology^71^; Nras is an oncogene that is connected to abnormal cranium morphology^72^; Hprt is linked to abnormal pharyngeal arch mesenchyme morphology^73^; Sirt1 is associated with abnormal palatal rugae morphology^74^.

#### Neuroblastoma tumor samples

The gene expression profiles of clinically high-risk (HR) neuroblastoma patients are compared to non-HR patients. The permutation test is performed using $B=100$ permutations, and the threshold for significance is set to 0.01. The top 10 results for DC pathways and DC genes are given in Supplementary Tables S3 and S4, respectively.

Several of the top pathways are directly involved in cell proliferation: “Mitotic Telophase/Cytokinesis” is involved in the pinching of the cell into two daughter cells during the final phase of mitosis; “Chk1/Chk2(Cds1) mediated inactivation of Cyclin B:Cdk1 complex” is an event that can occur during the monitoring of the genome for damage to prevent the transition into the next cell cycle; and “ERKs are inactivated” is a MAPK pathway that has a role in several fundamental cellular functions, including proliferation, cell survival, and apoptosis. A rewiring in any of these pathways might explain how a tumor becomes malignant or resistant to treatment in the high-risk group.

**Figure 4:**
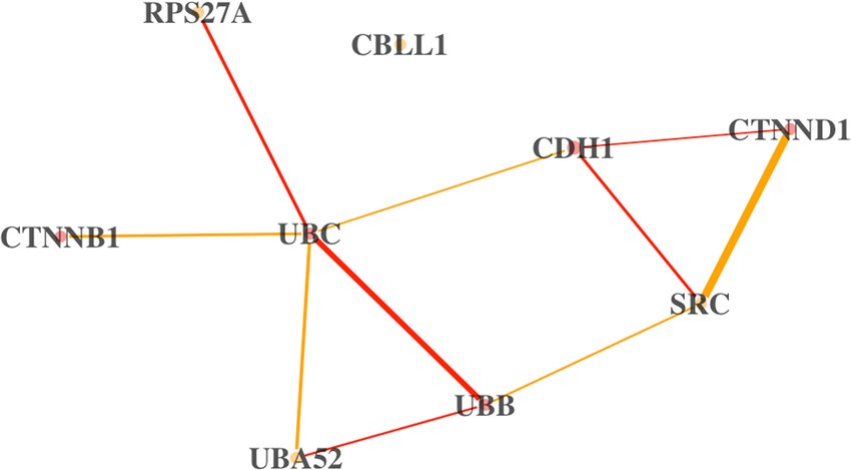
The differential network for the pathway “InlA-mediated entry of Listeria monocytogenes into host cells” in neuroblastoma tumors between HR and non-HR patients. Tan edges are associations that are stronger in non-HR patients, and red edges are stronger in HR patients. The edges are scaled proportionate to the DC score, and the nodes are scaled proportionate to the fold change in expression between the two groups. If the monotonized p-values are preferred, then two edges remain significant: SRC-CTNND1 and UBB-UBC.

The differential network for the pathway “InlA-mediated entry of Listeria monocytogenes into host cells” is shown in Fig. 4. This pathway contains the proto-oncogene SRC, which is also one of the top DC genes. The over-expression of this gene in colon cancer has been associated with accelerated metastatic growth and resistance to chemotherapeutic treatments^75^. One of the strongest differential connections in this pathway is between SRC and CTNND1 - the pair have a stronger connection in non-HR patients. It has been suggested that CTNND1 can modulate anchorage-independent growth induced by SRC^76^. This type of growth is a characteristic of metastatic potential^77^. However, it was suggested that the CTNND1 modulation may be reversed by the downstream ROCK cascade^76^. We checked to see if any pathways containing any ROCK genes were differentially connected and found one such pathway: “EPHB-mediated forward signaling.” The top differential connection in this pathway is between ROCK2 and EPHB6. Incidentally, a recent study suggests that treating certain breast cancer patients with SRC inhibitors may be more effective in the cases where EPHB6 is under-expressed^78^. We do not find differential expression in any of these genes in the neuroblastoma dataset, however the differential connectivity of EPHB6, ROCK2, and CTNND1 may be a sign of possible somatic mutations that are affecting their functionality and possibly impeding their ability to regulate SRC, resulting in metastatic growth.

Many of the top DC genes, shown in Supplementary Table S4, have a known relation to cancer. As for example, FADD is involved in the mediation of cell apoptotic signals, TMEM219 is a cell death receptor for IGFBP3, and TNFRSF10B is a receptor for apoptosis signaling. As demonstrated with SRC, by looking at the specific differential connections of these genes we may find a biological mechanism, novel to neuroblastoma, that explains the difference between the HR and non-HR patients.

## Discussion

A differential network analysis of gene-gene co-expression networks is used to explore the differences between the underlying biochemical processes of two groups. In this study, we propose a framework for incorporating knowledge of gene regulatory pathways into this type of analysis. The practitioner is able to choose any association measure that is deemed appropriate to address the question at hand. Differential connectivity scores are computed to find DC pathways, DC genes, and DC edges, using the proposed measure, *δ*_*E*_, and a monotonized permutation test provides p-values for these differential connectivity scores.

An exploratory analysis is demonstrated on two datasets, and in both examples we find biologically meaningful genes and pathways that are differentially connected. The utility of analyzing the co-expression networks at different levels is emphasized: DC pathways provide the biological context of the results, DC genes are possible candidates for further investigation, and DC edges identify the specific interactions that may motivate hypotheses to test. Furthermore, the results are automatically partitioned by pathway, so domain knowledge can be used to help select and sort the results that make most biological sense.

Incidentally, the penultimate version of this manuscript found Kat2b as a potential novel finding in craniofacial development. An article that was very recently published supports the validity of this finding^69^, which is the first study in the literature to describe the role of Kat2b in craniofacial development. The results of our analysis suggest this gene may also have a more specific role in the fusion of palatal shelves.

Another benefit of using pathways is the savings in computation time. In fact, many modern methods for differential network analysis have computational restrictions that implicitly require genes to be subset on pathways, or for most genes in an expression profile to be filtered out in some other way prior to analysis. The proposed framework formalizes this act of using pathway knowledge.

A simulation study shows that including pathway information gives comparable performance to no pathway information, even if the pathway information is incomplete. This result is important since our knowledge of pathways is continuously growing, and any pathway may be missing genes or contain extraneous ones. The simulation results suggest that even if the pathway information is imperfect, the differential network analysis is still able to find DC pathways, DC genes, and DC edges without compromising specificity or true discovery rate. A second simulation study considers the performance of four modern approaches. However, this study underlines the fact that different methods will typically use different notions of association. When two approaches are estimating different things, their performance is not directly comparable.

There are a few avenues for future research. Currently, pathways are treated and analyzed independently, but pathways are dependent and often have overlapping genes. For example, the Reactome pathways have a hierarchical structure; pathways with a general function are broken down into specialized events. If a gene is differentially connected due to a mutation, then perhaps it should be differentially connected in all pathways it’s involved in. Incorporating this kind of dependency may be a way to increase sensitivity.

Another concern is that by sub-setting on pathways, important genes that are not included in any pathway could be missed; these could be genes with no known functionality that lead to a novel discovery. One possible solution is to add a preliminary step in which the pathways are inferred from the data; an unsupervised clustering algorithm that allows for overlapping clusters may be able to approximate the known pathways while incorporating all of the genes that are expressed. To make this approach feasible for smaller samples, a semi-supervised approach that also incorporates known pathways could be devised. These ideas are left for future investigation.

## Supplementary information

Supplementary Materials
