# Supplementary material for: Integrating gene regulatory pathways into differential network analysis of gene expression data
Source: Sci Rep. 2019 Apr 2;9:5479. doi: 10.1038/s41598-019-41918-3 (PMC6445151; doi:10.1038/s41598-019-41918-3)
Supplement: Supplementary file 1 — Supplementary Materials [file 41598_2019_41918_MOESM1_ESM.pdf]

Supplementary Material for "Integrating gene regulatory pathways into differential network analysis of gene expression data"

Tyler Grimes[1], S. Steven Potter[2], and Somnath Datta[3,*]

[1,3]University of Florida, Department of Biostatistics, Gainesville, 32611, USA
[2]University of Cincinnati, Department of Pediatrics, Cincinnati, 45229, USA
[*]somnath.datta@ufl.edu

ABSTRACT

This supplement contains the algorithm for computing the monotonized p-values in the permutation testing procedure; tables of the top 10 differentially connected pathways and genes for the craniofacial and neuroblastoma datasets; and additional results from the simulation study.

S1 Monotonized p-values

The permutation testing procedure can be modified for multiple hypothesis testing[1,2]. Within a given pathway, we may be interested in testing the differential connectivity of a collection of m edge sets, $\mathscr{E} = \{E_i : i = 1, ..., m\}$. For example, E_i may contain all of possible connections to gene i, and it would correspond to the null hypothesis that connections in E_i are constant across groups. In this case, the set of hypotheses contained in $\mathscr{E}$ pertain to the differential connectivity of each gene in the pathway. When carrying out the standard permutation testing procedure on this set of hypotheses, we often find that the estimated p-values are not monotonic with respect to the original test statistics. That is, larger test statistics don't necessarily lead to smaller p-values.

This property of monotonicity can be enforced through a step-down p-value calculation, which is outlined here. These monotonized p-values will be more conservative than the usual permutation p-values. This helps control the overall experiment-wise error rate in the case of multiple hypothesis testing at the cost of lower sensitivity.

1. Compute $d_{i0} = \delta_{E_i}(\hat{S}^1, \hat{S}^2)$ on the original sample for $i = 1, ..., m$.
2. Find the rank orders, $r_1, ..., r_m$ such that $d_{r_1 0} > ... > d_{r_m 0}$.
3. Set $X = \begin{bmatrix} X^1 \\ X^2 \end{bmatrix} \in \mathbb{R}^{(n_1+n_2)\times p}$ and set $j = 1$.
4. Permute the rows of X to obtain a permuted matrix X^*. Use the first n_1 rows for X^{1*} and the remaining n_2 rows for X^{2*}.
5. Estimate the association networks $\hat{S}^{1*}$ and $\hat{S}^{2*}$ using the permuted samples X^{1*} and X^{2*}.
6. Compute $d_{ij} = \delta_{E_i}(\hat{S}^{1*}, \hat{S}^{2*})$ for $i = 1, ..., m$.
7. Monotonize the permuted test statistics with respect to the original rank orders by
   - 7.1. $d^*_{mj} = d_{r_m j}$
   - 7.2. $d^*_{(m-1)j} = \max(d_{r_{(m-1)} j}, d^*_{mj})$
   - ...
   - 7.m. $d^*_{1j} = \max(d_{r_1 j}, d^*_{2j})$
8. Increment $j = j + 1$.
9. Repeat steps 4-8 for a total of B times.

10. Compute $p_{r_i} = (B+1)^{-1}(\sum_{j=1}^{B} I(d_{r_i 0} \leq d^*_{ij}) + 1)$ for $i = 1, ..., m$.

11. Monotonize the estimated p-values by

    11.1. $p^*_{r_1} = p_{r_1}$

    11.2. $p^*_{r_2} = \max(p_{r_2}, p^*_{r_1})$

    ...

    11.m. $p^*_{r_m} = \max(p_{r_{(m-1)}}, p^*_{r_m})$

12. Return $p*$ as the monotonized p-values.

S2 Craniofacial results

Table S1. Differentially connected pathways between the anterior and posterior domains of each compartment. The top 10 DC pathways in each compartment are shown with: the Reactome pathway name; the DC score; the number of genes in the pathway, and, of those, the number that are significantly DC (in parenthesis); the mean expression level of the gene in each region, expressed as fold change over the average mean of all genes; and the p-value for the t-test of differential expression (DE) of the pathway between the regions. These DE p-values are provided to demonstrate that a gene may be DC without being DE.

Reactome pathway	DC score	Num. of genes	FC anterior	FC posterior	DE p-value
**Lateral**					
Gap junction degradation	0.20	10(0)	1.31	1.35	0.097
Association of licensing factors with the pre-replicative complex	0.13	15(0)	1.24	1.27	0.0056
Signaling by NOTCH1	0.13	16(1)	1.22	1.18	0.0496
Deadenylation of mRNA	0.08	25(0)	1.18	1.20	0.0348
IRAK2 mediated activation of TAK1 complex	0.21	10(0)	1.45	1.44	0.703
Establishment of Sister Chromatid Cohesion	0.18	11(0)	1.18	1.20	0.757
p75NTR recruits signalling complexes	0.16	13(0)	1.28	1.30	0.474
Gap junction trafficking and regulation	0.14	15(0)	1.18	1.20	0.211
Sema3A PAK dependent Axon repulsion	0.13	15(0)	1.09	1.08	0.502
Ephrin signaling	0.13	15(0)	1.12	1.09	0.175
**Medial**					
Processive synthesis on the C-strand of the telomere	0.19	11(1)	1.02	1.05	0.653
Removal of the Flap Intermediate	0.15	14(0)	1.00	1.04	0.464
A tetrasaccharide linker sequence is required for GAG synthesis	0.08	26(1)	1.04	0.99	0.440
HS-GAG biosynthesis	0.08	26(0)	1.03	0.95	0.359
**Nasal**					
Regulation of HSF1-mediated heat shock response	0.13	16(1)	1.30	1.28	0.0958
VEGFR2 mediated vascular permeability	0.09	23(0)	1.11	1.04	0.0226
Mitotic Telophase/Cytokinesis	0.13	14(2)	1.30	1.31	0.814
PCNA-Dependent Long Patch Base Excision Repair	0.13	17(1)	1.09	1.17	0.243
Deadenylation of mRNA	0.09	25(0)	1.21	1.24	0.576
**Oral**					
Removal of the Flap Intermediate	0.14	14(0)	1.05	1.06	0.940
Polymerase switching on the C-strand of the telomere	0.13	14(0)	1.09	1.10	0.908
Lagging Strand Synthesis	0.10	20(0)	1.07	1.08	0.941
Downregulation of ERBB2 signaling	0.08	26(1)	0.98	1.03	0.801
Smooth Muscle Contraction	0.08	27(1)	1.11	1.03	0.629

Table S2. DC genes between anterior and posterior domains of each compartment. 447 genes are significantly DC; 81 of those have average expression level in the bottom quartile and are removed. The top 10 remaining DC genes are shown with: the MGI symbol of the gene; the DC score, δ_E; the number of significant pathways the gene is in, and, of those, the number of pathways it is significantly DC in (in parenthesis); the mean expression level of the gene in each region, expressed as fold change over the average mean of all genes; and the p-value for the t-test of differential expression.

MGI symbol	DC score	Num. of pathways	FC anterior	FC posterior	DE p-value
**Lateral**					
Atp2b4	0.05	6(1)	1.18	1.02	0.0401
Ep300	0.08	26(1)	1.34	1.33	0.391
Ngfr	0.08	8(1)	1.71	1.77	0.580
Stat3	0.07	10(1)	1.22	1.20	0.231
Dbnl	0.06	6(1)	1.23	1.29	0.543
Nras	0.06	32(1)	1.54	1.49	0.295
Nudc	0.05	2(1)	1.46	1.51	0.191
Hprt	0.03	3(1)	1.24	1.29	0.338
Sirt1	0.02	4(1)	1.03	1.05	0.483
Atf1	0.01	8(2)	1.74	1.75	0.757
**Medial**					
Fyn	0.07	32(3)	1.12	1.24	0.0477
Stat1	0.06	9(1)	1.05	1.00	0.0909
B3glct	0.06	2(1)	0.95	1.18	0.0185
Psen1	0.05	11(2)	1.04	1.09	0.0152
Acaa2	0.01	1(1)	1.18	1.36	0.0661
Rpa1	0.06	42(1)	1.22	1.23	0.726
Dlg1	0.05	4(1)	1.21	1.20	0.293
Sdc1	0.04	7(2)	1.42	1.33	0.520
Rbp1	0.04	1(1)	1.19	1.38	0.100
Ctnnb1	0.03	20(1)	1.82	1.86	0.984
**Nasal**					
Wnt5a	0.06	7(1)	1.72	1.11	0.00107
Pik3r1	0.05	46(1)	1.39	1.15	0.0662
Gm11808	0.03	159(5)	2.39	2.54	0.0159
Cpsf4	0.03	4(1)	0.94	1.12	0.064
Arrb2	0.03	6(2)	1.01	1.10	0.0979
Ado	0.02	1(1)	1.07	1.02	0.086
Mcm3	0.01	7(1)	1.36	1.53	0.0705
Txnip	0.07	3(1)	1.27	1.25	0.664
Atf2	0.05	8(1)	1.89	1.58	0.227
Plk1	0.05	13(1)	1.20	1.34	0.299
**Lateral**					
Ino80d	0.02	4(2)	1.39	1.25	0.00104
Ngfr	0.07	8(1)	2.06	1.85	0.207
Krt5	0.04	3(1)	1.11	0.80	0.148
Cry1	0.04	1(1)	1.04	0.96	0.536
Sri	0.03	7(1)	1.50	1.42	0.354
Cnot6	0.03	4(1)	1.33	1.28	0.581
Ppp2cb	0.02	31(1)	1.47	1.42	0.807
Prkar2b	0.02	13(1)	1.14	1.11	0.782
Tmed5	0.02	1(1)	1.68	1.60	0.991
Cald1	0.01	1(1)	1.90	1.75	0.903

S3 Neuroblastoma results

Table S3. DC pathways between clinically HR and non-HR neuroblastoma patients. The top 10 DC pathways are shown with: the Reactome pathway name; the DC score; the number of genes in the pathway, and, of those, the number that are significantly DC (in parenthesis); the mean expression level of the pathway for each group, expressed as the fold change from the average mean expression across all pathways; and the p-value for the t-test of differential expression.

Pathway	DC score	Num. of genes	FC High-risk	FC Normal	DE p-value
Mitotic Telophase/Cytokinesis	0.17	14(1)	1.19	1.15	1.31e-21
InlA-mediated entry of Listeria monocytogenes into host cells	0.16	10(3)	1.28	1.27	0.00365
ATF6 (ATF6-alpha) activates chaperone genes	0.16	10(1)	1.26	1.24	1.57e-15
Translocation of ZAP-70 to Immunological synapse	0.16	19(4)	1.02	1.12	1.49e-06
Signaling by NOTCH1 HD Domain Mutants in Cancer	0.16	15(1)	1.21	1.22	0.000504
Removal of the Flap Intermediate	0.16	14(1)	1.17	1.12	7.32e-31
Nucleobase biosynthesis	0.16	15(1)	1.15	1.11	2.78e-28
Membrane binding and targetting of GAG proteins	0.15	15(3)	1.25	1.24	1.2e-12
Chk1/Chk2(Cds1) mediated inactivation of Cyclin B:Cdk1 complex	0.15	14(1)	1.2	1.17	1.4e-14
ERKs are inactivated	0.15	13(2)	1.18	1.19	0.332

Table S4. Differentially connected genes between clinically high-risk versus normal neuroblastoma patients. The top 10 significant DC genes (using the monotonized p-values) are shown with: the HGNC symbol of the gene; the DC score; the number of pathways containing the gene, and, of those, the number of pathways that are significantly DC (in parenthesis); the mean expression level of the gene for each group, expressed as the fold change from the average mean expression across all genes; and the p-value for the t-test of differential expression.

HGNC symbol	DC score	Num. of pathways	FC High-risk	FC Normal	DE p-value
FADD	0.07	11(4)	1.21	1.15	4.04e-23
TMEM219	0.05	2(1)	1.20	1.20	1.45e-05
VPS28	0.05	2(2)	1.42	1.43	9.25e-09
DUSP3	0.05	8(2)	1.33	1.36	0.00296
NFYC	0.05	6(2)	1.19	1.21	7.09e-16
DUSP4	0.05	10(2)	1.12	0.98	1.04e-07
TNFRSF10B	0.04	8(1)	1.13	1.04	1.95e-18
SRC	0.04	50(3)	1.24	1.22	4.43e-06
MIB2	0.04	4(1)	1.21	1.27	4.23e-12
ST3GAL6	0.04	6(1)	1.20	1.18	0.000831

S4 Simulation

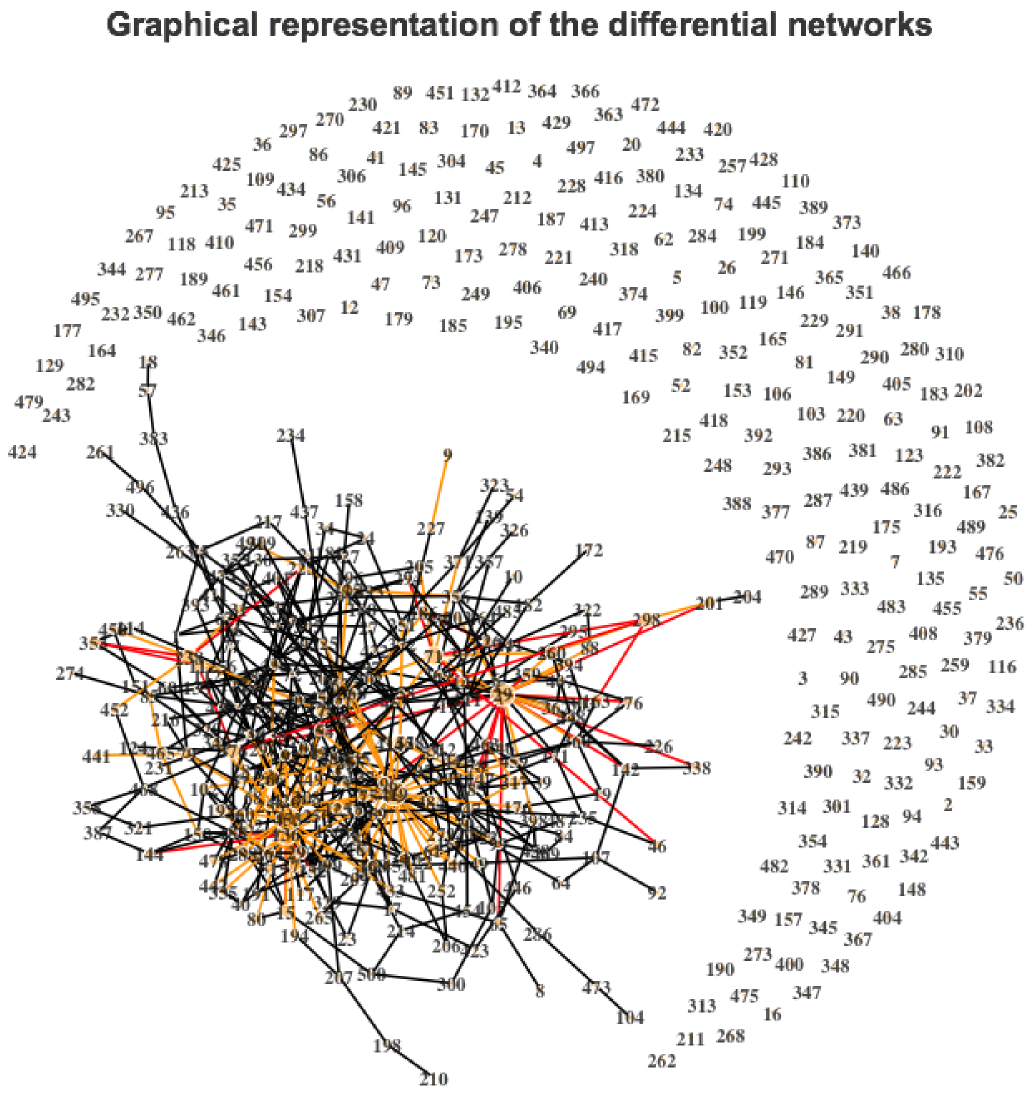

Figure S1. The underlying network used in the simulation. Black edges indicate connections common to both groups, brown edges are those only in the first group, and red edges are those only in the second.

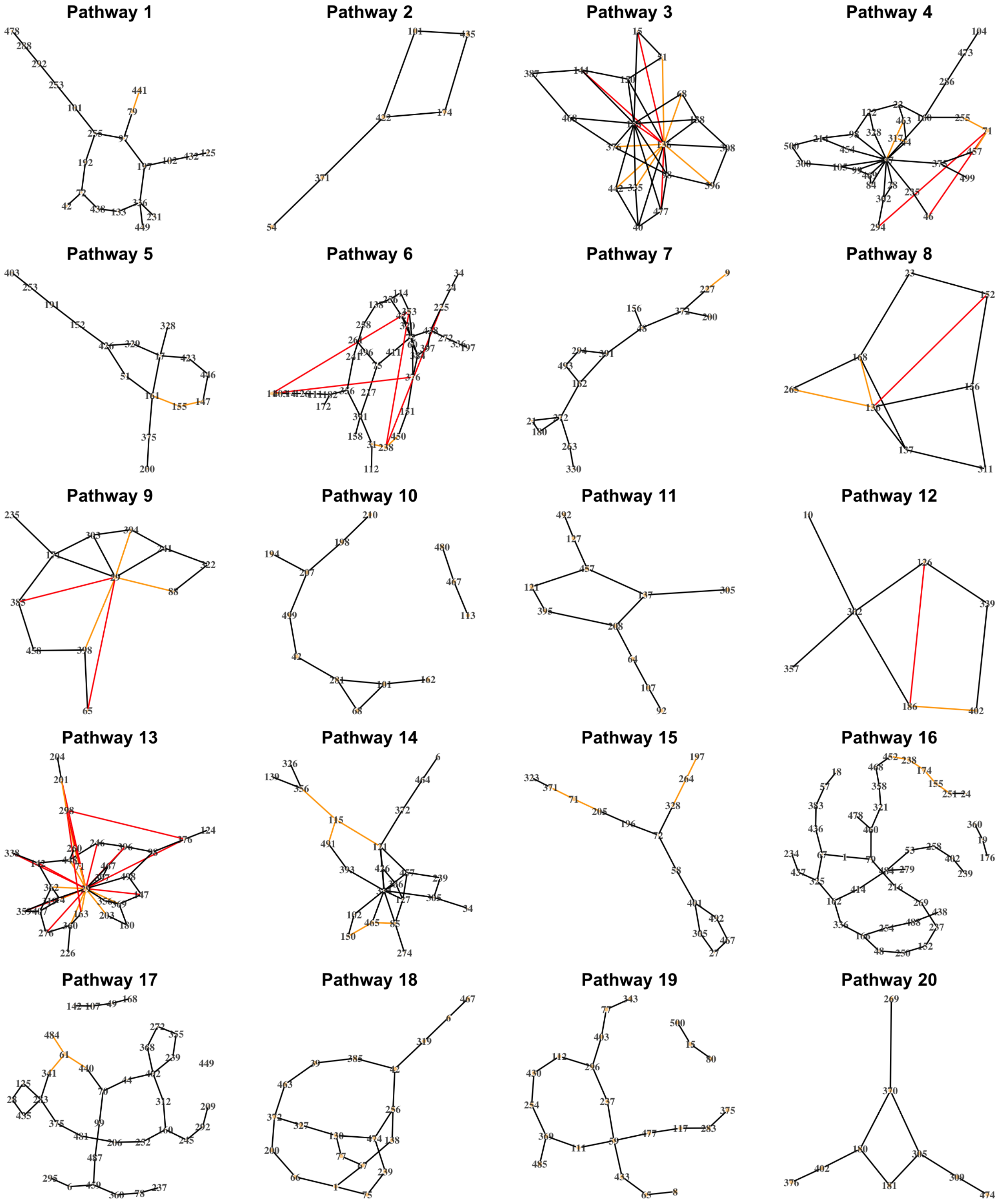

Figure S2. The underlying network used in the simulation is composed of these 20 overlapping pathways. Black edges indicate connections common to both groups, brown edges are those only in the first group, and red edges are those only in the second. Note that each pathway has a varying degree of differential connectivity; a small number of pathways have big changes in their network, for example if it contains a perturbed hub gene, while others only have small or no changes in connectivity.

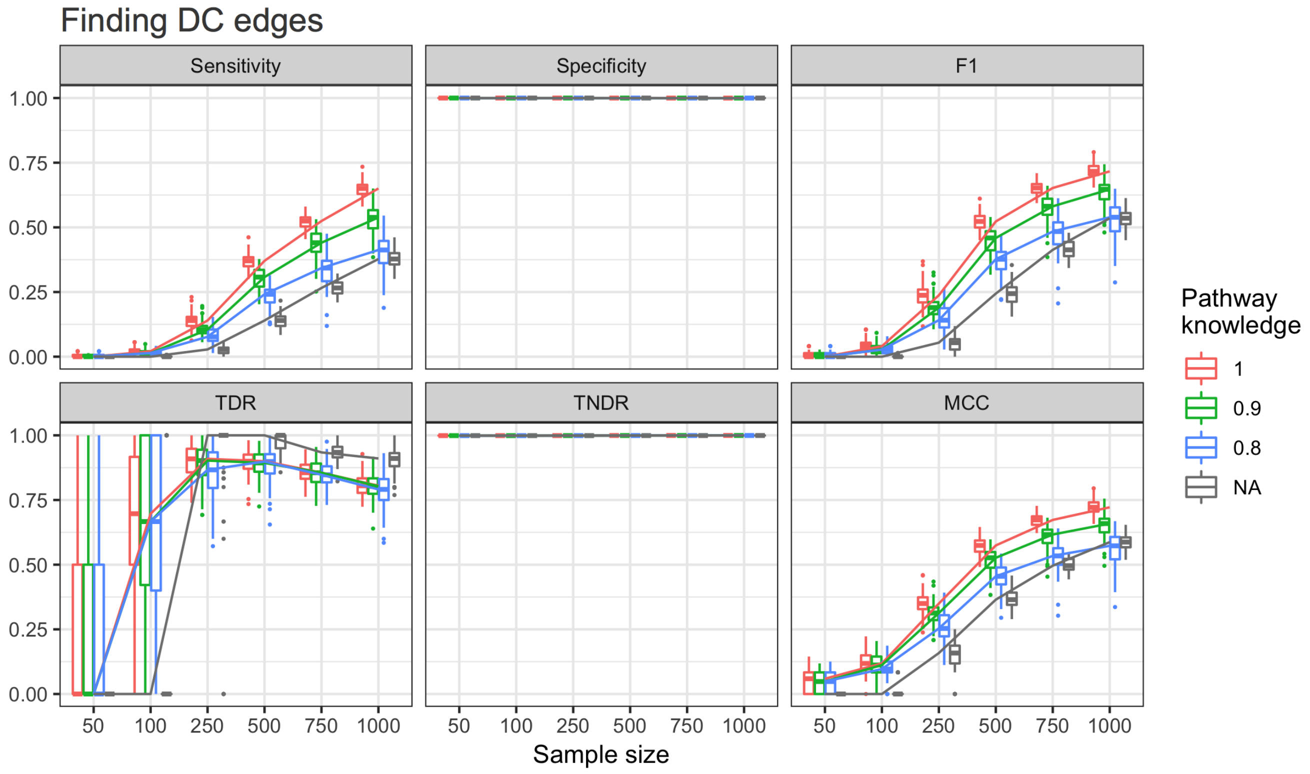

Figure S3. Simulation results from 100 generated datasets, testing for DC edges using Pearson correlations. The permutation tests are performed using 100 permutations and a significance threshold of 0.05 for the monotonized p-values. Results are shown for the differential network analysis conducted without pathway information (black), with complete pathway information (red), with 90% correct pathway information (green), and 80% correct pathway information (blue). For each measure, higher values indicate better performance.

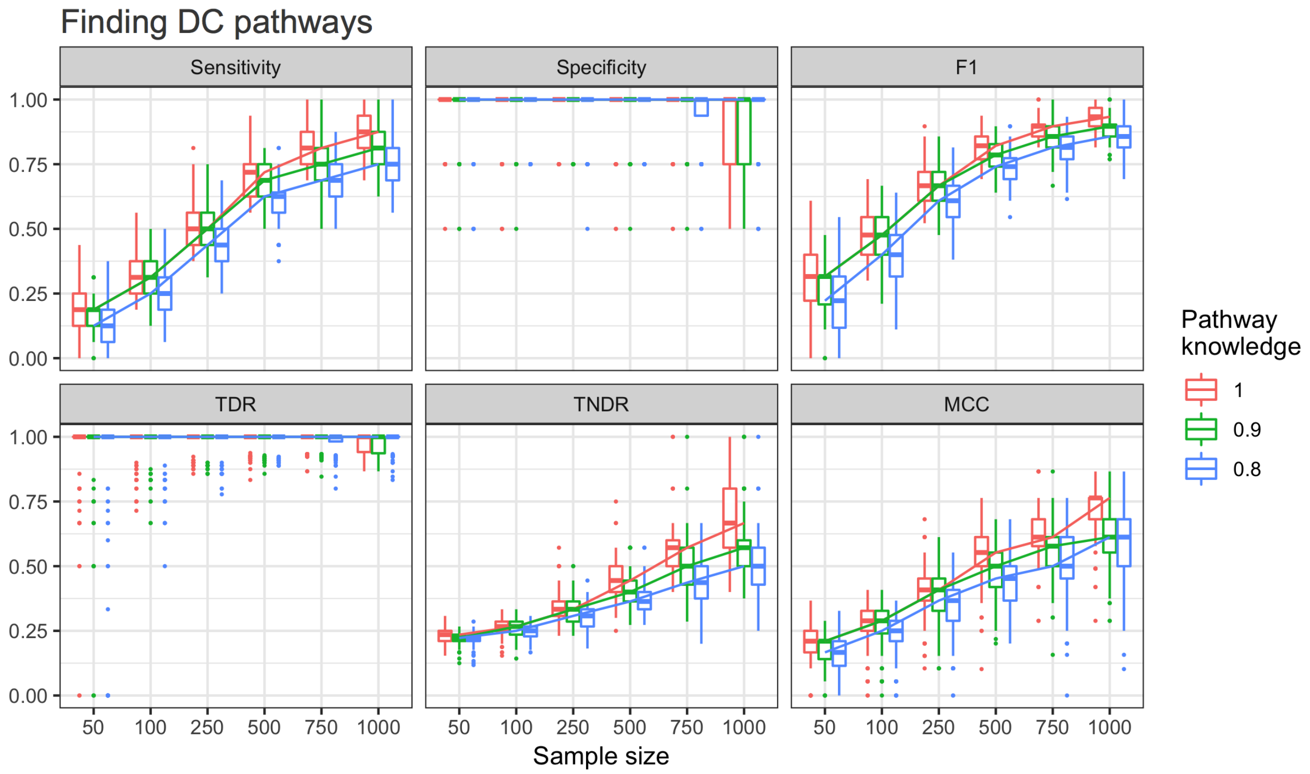

Figure S4. Simulation results from 100 generated datasets, testing for DC pathways using partial correlations and an L_2 norm for the DC scores. The permutation tests are performed using 100 permutations and a significance threshold of 0.05. Results are shown for the differential network analysis conducted with complete pathway information (red), with 90% correct pathway information (green), and 80% correct pathway information (blue). For each measure, higher values indicate better performance.

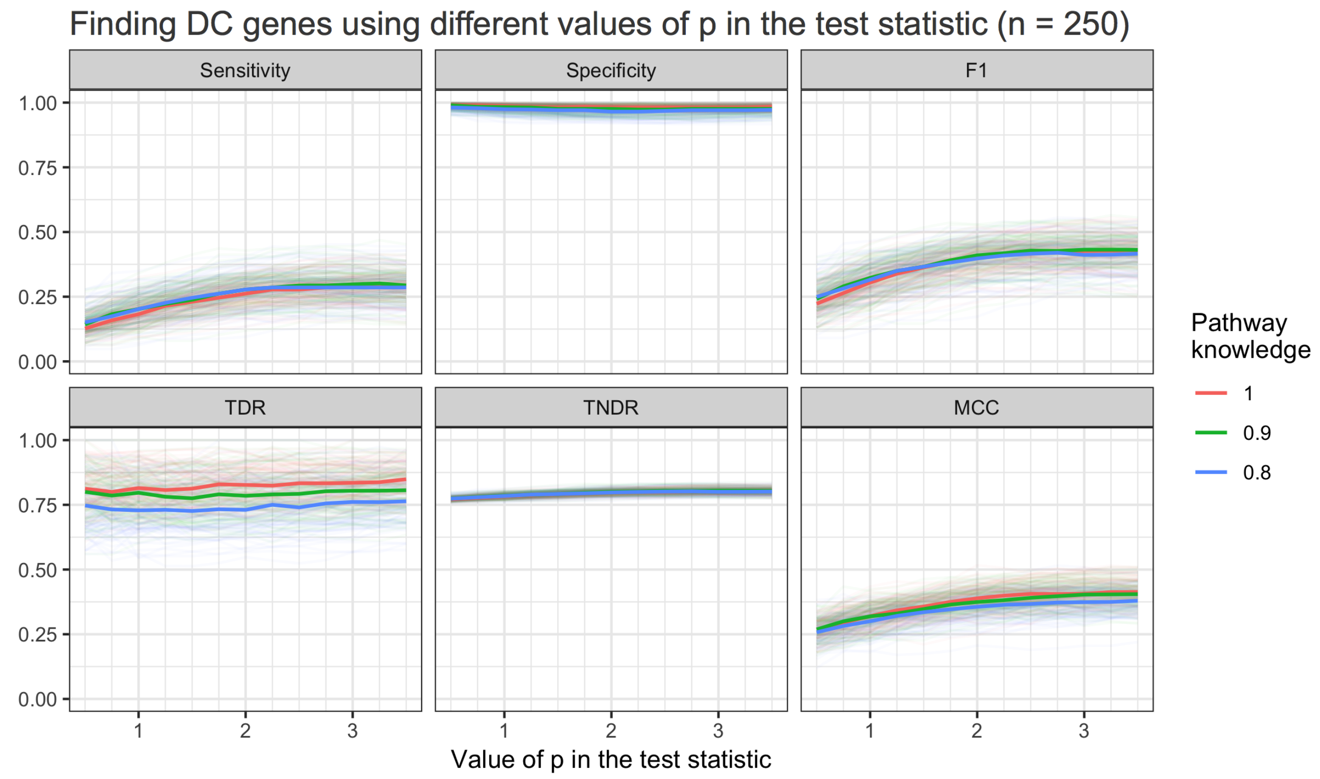

Figure S5. Simulation results for various choices of p from 100 generated datasets with a sample size of $n = 250$, testing for DC genes using partial correlations. The permutation tests are performed using 100 permutations and a significance threshold of 0.05 for the monotonized p-values. Results are shown for the differential network analysis conducted with complete pathway information (red), with 90% correct pathway information (green), and 80% correct pathway information (blue). For each measure, higher values indicate better performance.

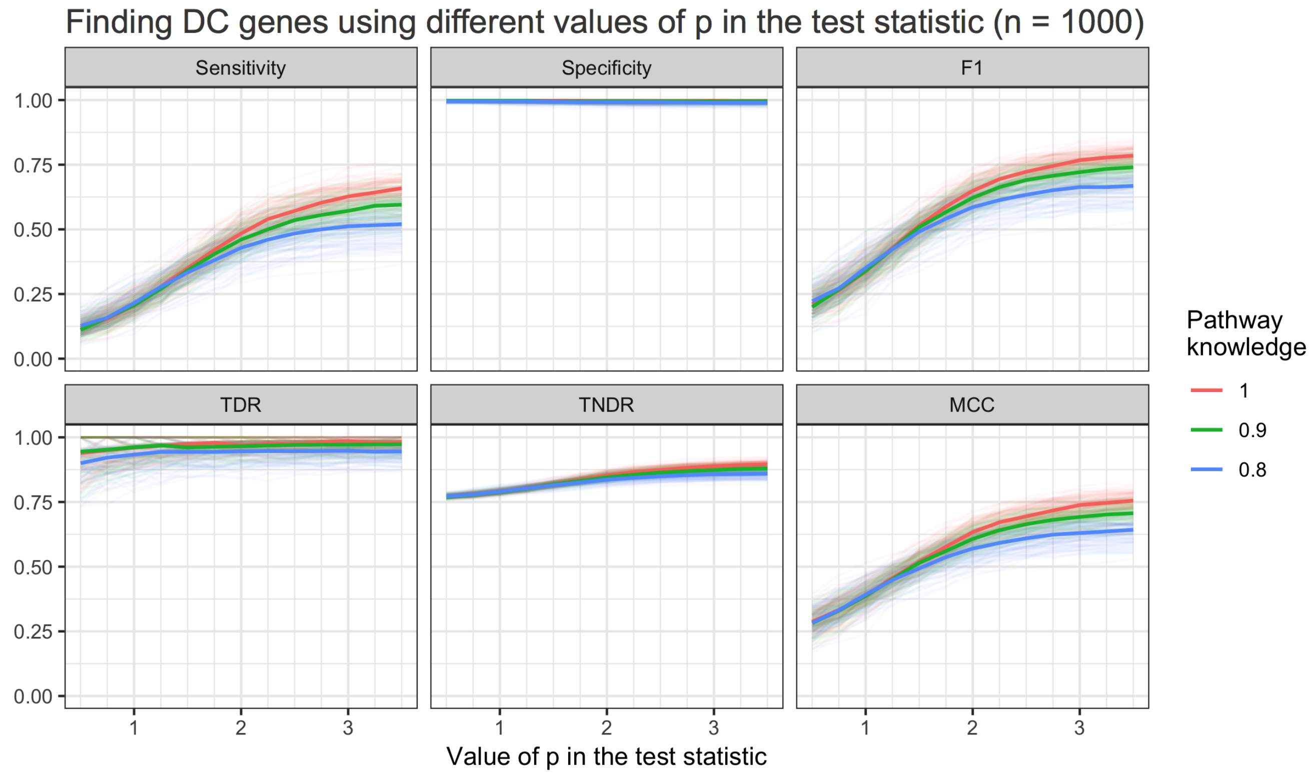

Figure S6. Simulation results for various choices of p from 100 generated datasets with a sample size of $n = 1000$, testing for DC genes using partial correlations. The permutation tests are performed using 100 permutations and a significance threshold of 0.05 for the monotonized p-values. Results are shown for the differential network analysis conducted with complete pathway information (red), with 90% correct pathway information (green), and 80% correct pathway information (blue). For each measure, higher values indicate better performance.

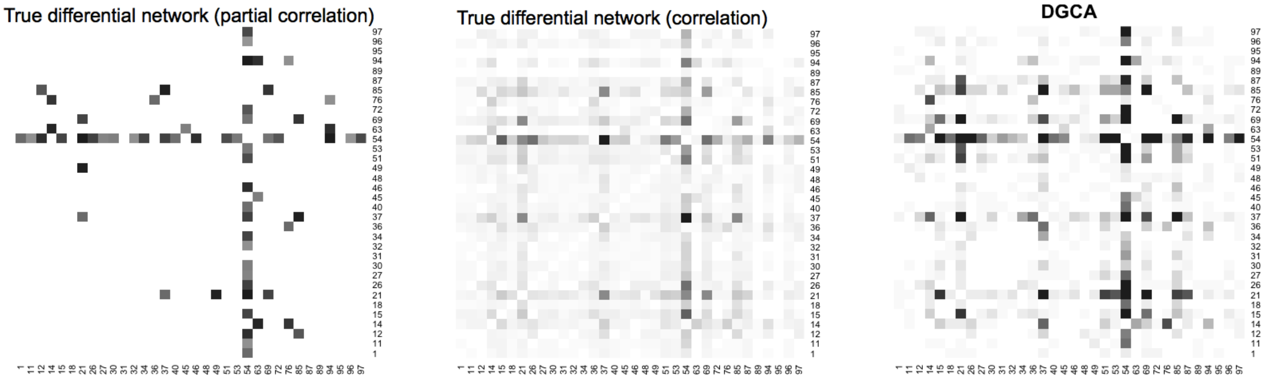

Figure S7. The true differential network using partial correlation as the measure of association (left), Pearson's correlation coefficient (middle), and the average differential networks estimated by DGCA based on 20 generated samples of size $n = 250$ (right). Since DGCA is based on correlation, it should be compared to the middle network for a fair assessment of its performance. In this case, we see that many of the differential edges that look like false discoveries when compared to the partial correlation network (left) actually are true discoveries of the Pearson's correlation network (middle).

References

1. Westfall, P. H. & Young, S. S. *Resampling-based Multiple Testing: Examples and Methods for p-value Adjustment* (Wiley-Interscience, 1993).

2. Datta, S. *et al.* An empirical bayes adjustment to increase the sensitivity of detecting differentially expressed genes in microarray experiments. *Bioinformatics* **20**, 235–242 (2004).
